# Response to: Comment on “Choroidal Thickness in Patients with Mild Cognitive Impairment and Alzheimer's Type Dementia”

**DOI:** 10.1155/2016/5496356

**Published:** 2016-10-17

**Authors:** Mehmet Bulut, Aylin Yaman, Muhammet Kazim Erol, Fatma Kurtuluş, Devrim Toslak, Berna Doğan, Deniz Turgut Çoban, Ebru Kaya Başar

**Affiliations:** ^1^Antalya Training and Research Hospital, Ophthalmology Department, 07050 Antalya, Turkey; ^2^Antalya Training and Research Hospital, Neurology Department, 07050 Antalya, Turkey; ^3^Department of Animal Science Biometry and Genetics Unit, Faculty of Agriculture, Akdeniz University, 07070 Antalya, Turkey

In our study, we found out that choroidal thickness (CT) decreased in the eyes of patients with both Alzheimer's type dementia (ATD) and mild cognitive impairment (MCI) compared to the healthy control group. Therefore, we suggested that CT value could be used as a new biomarker in early diagnosis of ATD and MCI patients and follow-up of their progression [1].

We would like to thank Uzun S. for the valuable comments and contributions for our paper [2]. These comments were useful for us. Choroidal thickness (CT) values may vary during the day and on different days [3]. As we specified in our study, we performed optical coherence tomography (OCT) measurements on all participants on minimum 3 different days and 3 times consecutively in each measurement in order to ensure the lowest variation in CT values. We used the average of the measurements in our study. All measurements were performed from 9 a.m. to 11 a.m.

The systemic physiological and pathological conditions of people may also influence CT values [3]. Recent studies showed that smoking and coffee decreased CT [4, 5]. Also some systemic diseases like cardiovascular disease, diabetes mellitus, and systemic arterial hypertension may affect CT [6]. Furthermore, sildenafil and pseudoephedrine-like drugs cause an increase in choroidal circulation and thickness [7]. As we mentioned in our study, to ensure minimum impact on CT data, participants with diabetes mellitus, cardiovascular disease, systemic arterial hypertension, and other serious chronic systemic diseases were excluded from our study. Furthermore, smokers were also excluded. All participants were instructed not to consume caffeine in 12 hours before the measurements. Moreover, patients who were taking sildenafil and pseudoephedrine-like systemic drugs, which might affect CT values, were not included in the study. OCT measurements of all participants were performed in a very cozy room where the light levels were adjusted. Using all the above-mentioned criteria, we tried to make sure that there was minimum impact on CT values.

Finally, since we could not definitively detect how long some of the ATD and MCI patients had had the disease at the time of the first diagnosis, the correlation between the duration of the disease and CT was not analyzed in either ATD patients or MCI patients.

## References

[1] Bulut M., Yaman A., Erol M. K. (2016). Choroidal thickness in patients with mild cognitive impairment and Alzheimer's type dementia. *Journal of Ophthalmology*.

[2] Uzun S. (2016). Comment on ‘choroidal thickness in patients with mild cognitive impairment and Alzheimer's type dementia’. *Journal of Ophthalmology*.

[3] Nickla D. L., Wallman J. (2010). The multifunctional choroid. *Progress in Retinal and Eye Research*.

[4] Sızmaz S., Küçükerdönmez C., Pınarcı E. Y., Karalezli A., Canan H., Yılmaz G. (2013). The effect of smoking on choroidal thickness measured by optical coherence tomography. *British Journal of Ophthalmology*.

[5] Vural A. D., Kara N., Sayin N., Pirhan D., Ersan H. B. A. (2014). Choroidal thickness changes after a single administration of coffee in healthy subjects. *Retina*.

[6] Wong I. Y., Wong R. L., Zhao P., Lai W. W. (2013). Choroidal thickness in relation to hypercholesterolemia on enhanced depth imaging optical coherence tomography. *Retina*.

[7] Kim D. Y., Silverman R. H., Chan R. V. P. (2013). Measurement of choroidal perfusion and thickness following systemic sildenafil (Viagra). *Acta Ophthalmologica*.

